# 

**Published:** 1858-03

**Authors:** 


### CONTENTS.

ORIGINAL COMMUNICATIONS— PAGE.

Uses of the Ophthalmoscope. By E. L. Holmes, M.D. .... 101

Does Air enter the Uterine Veins during Labor, or shortly after, and Cause Death? By J. P. DeBruler, M.D. .... 114

Polypiform Concretions in the Cavities of the Heart, with a Case. By P. A. Allaire, M.D. .... 117

Enlargement of the Spleen. By J. W. Crooks, M.D. .... 123

Doctor Bacon's Case. By W. H. Byford, M.D. .... 127

BOOK NOTICES—

The Principles and Practice of Obstetrics. By Henry Miller, M.D. .... 132

A Practical Treatise on Diseases of Children. By J. Forsyth Meigs, M.D. .... 132

The Physician's Handbook of Practice and Memoranda for 1858. By Wm. Elmer, M.D., and Levi Reuben, M.D. .... 133

PROCEEDINGS OF MEDICAL SOCIETIES—

Meeting of the Henry County Medical Society .... 135

EDITORIAL—

Medical Education .... 136

Rush Medical College—Annual Commencement .... 142

Dr. D. D. Thompson, of Ottawa, Ill .... 143

Honey Bees as Diuretic .... 144

ADVERTISEMENTS .... 145, 146, 147, 148

